# Neurotransmitter networks in mouse prefrontal cortex are reconfigured by isoflurane anesthesia

**DOI:** 10.1152/jn.00092.2020

**Published:** 2020-04-29

**Authors:** Xiaoying Zhang, Aaron G. Baer, Joshua M. Price, Piet C. Jones, Benjamin J. Garcia, Jonathon Romero, Ashley M. Cliff, Weidong Mi, James B. Brown, Daniel A. Jacobson, Ralph Lydic, Helen A. Baghdoyan

**Affiliations:** ^1^Department of Anesthesiology, University of Tennessee Medical Center, Knoxville, Tennessee; ^2^Department of Psychology, University of Tennessee, Knoxville, Tennessee; ^3^Anesthesia and Operation Center, Chinese PLA General Hospital, Beijing, China; ^4^Office of Information Technology, University of Tennessee, Knoxville, Tennessee; ^5^Oak Ridge National Laboratory, Oak Ridge, Tennessee; ^6^Bredesen Center for Interdisciplinary Research and Graduate Education, University of Tennessee, Knoxville, Tennessee; ^7^Molecular Ecosystems Biology Department, Lawrence Berkeley National Laboratory, Berkeley, California

**Keywords:** acetylcholine, adenosine, liquid chromatography-dual mass spectrometry, loss of consciousness, machine learning

## Abstract

This study quantified eight small-molecule neurotransmitters collected simultaneously from prefrontal cortex of C57BL/6J mice (*n* = 23) during wakefulness and during isoflurane anesthesia (1.3%). Using isoflurane anesthesia as an independent variable enabled evaluation of the hypothesis that isoflurane anesthesia differentially alters concentrations of multiple neurotransmitters and their interactions. Machine learning was applied to reveal higher order interactions among neurotransmitters. Using a between-subjects design, microdialysis was performed during wakefulness and during anesthesia. Concentrations (nM) of acetylcholine, adenosine, dopamine, GABA, glutamate, histamine, norepinephrine, and serotonin in the dialysis samples are reported (means ± SD). Relative to wakefulness, acetylcholine concentration was lower during isoflurane anesthesia (1.254 ± 1.118 vs. 0.401 ± 0.134, *P* = 0.009), and concentrations of adenosine (29.456 ± 29.756 vs. 101.321 ± 38.603, *P* < 0.001), dopamine (0.0578 ± 0.0384 vs. 0.113 ± 0.084, *P* = 0.036), and norepinephrine (0.126 ± 0.080 vs. 0.219 ± 0.066, *P* = 0.010) were higher during anesthesia. Isoflurane reconfigured neurotransmitter interactions in prefrontal cortex, and the state of isoflurane anesthesia was reliably predicted by prefrontal cortex concentrations of adenosine, norepinephrine, and acetylcholine. A novel finding to emerge from machine learning analyses is that neurotransmitter concentration profiles in mouse prefrontal cortex undergo functional reconfiguration during isoflurane anesthesia. Adenosine, norepinephrine, and acetylcholine showed high feature importance, supporting the interpretation that interactions among these three transmitters may play a key role in modulating levels of cortical and behavioral arousal.

**NEW & NOTEWORTHY** This study discovered that interactions between neurotransmitters in mouse prefrontal cortex were altered during isoflurane anesthesia relative to wakefulness. Machine learning further demonstrated that, relative to wakefulness, higher order interactions among neurotransmitters were disrupted during isoflurane administration. These findings extend to the neurochemical domain the concept that anesthetic-induced loss of wakefulness results from a disruption of neural network connectivity.

## INTRODUCTION

Anesthetics once were thought to cause loss of wakefulness via a generalized inhibition of brain activity (Perouansky 2012). Now it has become clear that the brain remains electrically active during general anesthesia (Hudetz 2012) and that electrical activity patterns during surgical anesthesia are dynamic (Vlisides et al. 2019). Current theories concerning the mechanisms by which anesthetics cause loss of wakefulness focus on functional disconnection among brain regions (Alkire et al. 2008), loss of effective cortical connectivity (Hashmi et al. 2017), loss of coherence in the cortical electroencephalogram (Akeju et al. 2016), and a breakdown of information integration (Tononi et al. 2016). Most recently, decoupling between cortical pyramidal neurons has been suggested to play a key role in anesthetic-induced loss of consciousness (Suzuki and Larkum 2020). The foregoing findings have clinical relevance for intraoperative neurophysiological monitoring (Smith 2018), efforts to minimize the potential toxic effects of volatile anesthetics (Iqbal et al. 2019), and for their potential to address questions concerning the brain mechanisms generating states of consciousness (Baria et al. 2018; Kennedy and Norman 2005). Electrophysiology and neuroimaging provide important insights concerning altered network connectivity during general anesthesia, yet achieving a functional connectomics perspective of the brain (Fornito et al. 2015) must ultimately incorporate neurotransmitter data.

The canonical neuroscience perspective that “chemically mediated transmission is the major mode of neuronal communication” (Deutch 2013) supports the interpretation that neurotransmitter changes are lower level phenotypes mediating isoflurane-induced loss of wakefulness. Anesthetics alter membrane potential and release of neurotransmitters by direct actions on neurotransmitter receptors (Fong et al. 2017; Maldifassi et al. 2016) and ion channels (Covarrubias et al. 2015; Herold et al. 2017). The majority of in vivo studies that have quantified the effects of general anesthetics on neurotransmitter levels report data for only a single neurotransmitter (reviewed in Lydic et al. 2018; Müller et al. 2011). By contrast, the present study used in vivo microdialysis and liquid chromatography-dual mass spectrometry to quantify eight neurotransmitters collected simultaneously from mouse prefrontal cortex. These measures enabled a test of the hypothesis that isoflurane anesthesia differentially alters prefrontal cortex concentrations of neurotransmitters that have previously been shown to regulate wakefulness.

The prefrontal cortex was targeted because of its significant contribution to neurological diseases (Xu et al. 2019), the regulation of breathing (Herrero et al. 2018; Ramirez and Baertsch 2018), and cardiovascular function (Hassan et al. 2013; Wood et al. 2017). Isoflurane anesthesia was used as a tool to cause loss of wakefulness and to create a stable behavioral state during dialysis sample collection (Flint et al. 2010). The results show that concentrations of norepinephrine, adenosine, and dopamine in the dialysis samples were significantly greater during isoflurane anesthesia than during wakefulness. The concentration of acetylcholine was significantly lower during isoflurane anesthesia than during wakefulness, and concentrations of glutamate, serotonin, GABA, and histamine in the dialysis samples did not change. Evaluation of these data using machine learning algorithms led to two novel discoveries. First, neurotransmitter interactions observed during wakefulness were reorganized during isoflurane anesthesia. Second, states of wakefulness and isoflurane anesthesia could be differentiated based on neurotransmitter concentration profiles. The results from these state-of-the-art chemical and computational approaches signal a new direction for efforts to understand how multiple molecules and brain regions regulate wakefulness and the anesthesia-induced loss of wakefulness.

## MATERIALS AND METHODS

### 

#### Animals, drugs, and chemicals.

All experiments were approved by the University of Tennessee Institutional Animal Care and Use Committee and adhered to the *Guide for the Care and Use of Laboratory Animals* (National Research Council 2011). Twenty-three adult, male C57BL/6J (B6) mice (stock no. 000664) purchased from the Jackson Laboratory (Bar Harbor, ME) were used. Average (±SD) mouse body weight was 26.8 ± 1.6 g. All mice were maintained in good health throughout the study and were inspected daily by laboratory staff, as well as periodically by Office of Laboratory Animal Care veterinarians and staff. Mice were housed in a temperature- and humidity-controlled environment with ad libitum access to food and water. Lights went on at 8:00 AM and off at 8:00 PM. Isoflurane was purchased from Henry Schein (Indianapolis, IN). Ringer’s solution for perfusion of the dialysis probes, comprising 147.0 mM NaCl, 2.7 mM KCl, 1.2 mM CaCl_2_, and 0.85 mM MgCl_2_, was obtained from CMA Microdialysis (Holliston, MA).

#### Overview of the experimental design.

**Fig. 1.**
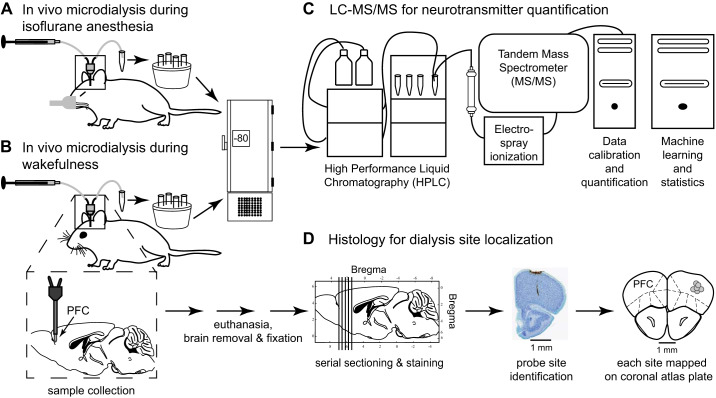
Workflow illustration of experimental design. In vivo microdialysis was used to collect 5 samples from each mouse during isoflurane anesthesia (*A*) or wakefulness (*B*). Enlargement schematizes placement of a dialysis membrane in the prefrontal cortex (PFC) during the sample collection period. Syringes perfused the dialysis probes with Ringer’s solution. Dialysis samples were collected in microcentrifuge tubes placed on ice and stored at −80°C until subsequent quantification of neurotransmitters (*C*) by high-performance liquid chromatography and tandem mass spectrometry (LC-MS/MS). Transmitter concentrations in the dialysis samples were analyzed using descriptive and inferential statistics, as well as machine learning approaches. *D*: mice were euthanized and brains were removed, fixed, sectioned, and stained. A representative histological section from this study is shown to illustrate how dialysis probe sites were localized. Coronal sections were matched with a mouse brain atlas (Paxinos and Franklin 2018) to localize each dialysis site and plot the midpoint of the dialysis membrane (gray circles) on the matching atlas plate.

Figure 1 provides a workflow illustration of the experimental design and methods. A between-subjects design was used to evaluate the two-tailed hypothesis that isoflurane anesthesia differentially alters neurotransmitter concentrations and interactions among transmitters in prefrontal cortex. Machine learning was applied to determine whether concentrations of specific neurotransmitters in dialysis samples obtained from the prefrontal cortex were predictive of behavioral state. Mice were tested either during administration of isoflurane (*n* = 11) or during wakefulness (*n* = 12), and each mouse was used for only one experiment. The primary outcome measure was neurotransmitter concentration. Intracranial guide tubes were implanted stereotaxically at least 1 wk before mice were used for a microdialysis experiment. Collection of dialysis samples began 30–40 min after insertion of the dialysis probe into the brain. After the last dialysis sample was collected, the probe was removed from the brain and mice were returned to their home cages. The isoflurane experiments were conducted first to provide data for evaluating analyte stability across time.

#### Surgical implantation of guide tubes for microdialysis.

Humane procedures for implantation of intracranial guide tubes, placement of microdialysis probes, and in vivo microdialysis (Coleman et al. 2006; Van Dort et al. 2009; Wathen et al. 2012) have been described in detail and are outlined below. All anesthetic inductions used loss of righting response as a surrogate measure for loss of consciousness (Vanini et al. 2014). Mice were placed in an induction chamber, and isoflurane (2.5%) was delivered in 100% oxygen at a flow rate of 1 L/min. When the mouse stopped moving, the chamber was gently tipped to turn the mouse on its side and to determine whether the mouse would right itself. When the mouse did not right itself, the mouse was removed from the induction chamber and isoflurane was delivered using a nose cone while the mouse was shaved and scrubbed for surgery. Antinociception was assessed using paw withdrawal in response to pinch of a hindlimb footpad. The mouse was then moved to a stereotaxic frame (David Kopf Instruments; model 962) equipped with a mouse adaptor (model 962) and a mouse anesthesia mask (model 907). Delivered isoflurane concentration was reduced to 1.5% at a flow rate of 0.6 L/min and was measured continuously by spectrophotometry (Cardiocap/5; Datex-Ohmeda). During surgical implantation of a microdialysis guide cannula (CMA 7), core body temperature was held at 36–37°C by use of a circulating hot water pump (TP400 T/Pump heat therapy system; Gaymar) connected to a water-filled heating pad. Delivered isoflurane concentration, core body temperature, and breathing rate were recorded every 10 min. A CMA 7 guide cannula was implanted 1 mm above the targeted dialysis site, which was 3 mm anterior, 1.6 mm lateral, and 2 mm ventral to bregma (Paxinos and Franklin 2018). At the conclusion of the guide cannula implantation surgery, isoflurane delivery was terminated and the mouse was placed on its back in a heated recovery chamber. When the mouse righted itself, it was again placed on its back and a second successful righting response was confirmed. When the mouse ambulated normally, it was returned to its home cage. Mice were given at least 1 wk of recovery from the guide tube implantation surgery before they were used for a microdialysis experiment.

#### In vivo microdialysis for sample collection.

In vivo microdialysis is an approach that permits the collection of signaling molecules from specific brain regions of behaving animals. Microdialysis collects molecules from the extracellular space where volume transmission is a key component of cellular communication in the brain (Marcoli et al. 2015). Volume transmission refers to neurotransmitters in the extracellular space, where they flow to distance sites and activate receptors located outside of the synapse (Fuxe et al. 2010).

For collection of dialysis samples during administration of isoflurane (Fig. 1*A*), mice were anesthetized as described above. Delivered isoflurane concentration was targeted at 1.3%, which is the EC_50_ for B6 mouse (Sonner et al. 2000), and was measured continuously by spectrophotometry. Isoflurane was delivered in 100% oxygen at a flow rate of 0.6 L/min. Delivered isoflurane concentration, core body temperature, and breathing rate were recorded every 12.5 min, as described above. Core body temperature and breathing rate remained stable and within physiological range throughout the period of dialysis sample collection. These end points were used to adjust delivered isoflurane concentration such that each mouse was held at similar levels of anesthesia throughout collection of the microdialysis samples. For dialysis sample collection during wakefulness (Fig. 1*B*), a microdialysis probe was inserted into the permanently implanted guide tube, without the use of anesthesia.

CMA 7 dialysis probes (Cuprophane membrane, 1 mm × 0.24 mm, 6-kDa cutoff) were perfused continuously (1.0 µL/min) with Ringer’s solution. The average start time for dialysis sample collection was 11:00 AM. Five sequential dialysis samples (25 µL each) were collected on ice from each mouse. When the last dialysis sample was collected, the probe was removed from the brain and mice in the isoflurane group recovered from anesthesia as described above before being returned to their home cages. Mice in the wakefulness group were observed for normal behavior after probe removal, before being placed back in their home cages. Dialysis samples were stored at −80°C for offline quantification (Fig. 1, *A* and *B*).

#### Neurotransmitter quantification.

Liquid chromatography and tandem mass spectrometry were used for detection and quantification of eight simultaneously collected neurotransmitters (Fig. 1*C*). An internal standard solution containing known concentrations of stable labeled isotopes of each analyte was added to every dialysis sample to determine the concentration of each endogenous neurotransmitter in every dialysis sample. The ability to measure multiple simultaneously collected neurotransmitters permitted novel analyses of interactions among those transmitters during states of wakefulness and isoflurane anesthesia. Offline quantification of neurotransmitter concentrations was performed by Charles River Laboratories, Inc. (South San Francisco, CA) using previously published methods (Bredewold et al. 2015; Flik et al. 2015) described below. Individuals who measured neurotransmitter concentrations were blinded to treatment conditions.

Analysis of norepinephrine, dopamine, serotonin, histamine, glutamate, and GABA was performed by adding 4 μL of internal standard solution (containing known concentrations of stable labeled isotopes of the analytes of interest) to 15 μL of experimental sample. The resulting mixture was derivatized online with 30 μL of SymDaQ reagent, and 30 μL were injected into the liquid chromatography system (Shimadzu Prominence series, Shimadzu, Japan) by an automated sample injector (SIL-20ACHT; Shimadzu). The above analytes were separated by liquid chromatography using a linear gradient of mobile phase B at a flow rate of 0.300 mL/min on a reversed-phase Phenomenex Synergi max-RP C12 column (3.0 × 100 mm, 2.5-μm particle size; Phenomenex) held at a temperature of 35°C. Mobile phase A consisted of ultra-purified H_2_O with 0.2% acetonitrile (ACN) and 0.1% formic acid (FA). Mobile phase B was 70% ACN, 30% ultra-purified H_2_O, and 0.1% FA. Postcolumn, the flow was mixed with a continuous additional mobile phase containing 90% ACN, 5% ultra-purified H_2_O, 5% methanol, and 0.1% FA + 10 mM ammonium formate at a flow rate of 0.15 mL/min. Acquisitions were achieved in positive ionization mode using an API 4000 triple quadrupole (Applied Biosystems) equipped with a TurboIonSpray interface. The ion spray voltage was set at 3.0 kV and the probe temperature was 200°C. The collision gas (nitrogen) pressure was held at 3 psi. Data were calibrated and quantified using the Analyst data system (Applied Biosystems; version 1.4.2).

To quantify acetylcholine, samples (5 µL) were mixed with 9 µL of internal standard (acetylcholine-d9), and 5 µL of the mixture were injected into the liquid chromatography-dual mass spectrometry system by an automated sample injector (SIL-20ACHT; Shimadzu). Chromatographic separation was performed on an ion exchange (150 × 2.1 mm, 5 µm) analytical column (Thermo Scientific BioBasic SCX; Keystone) held at a temperature of 30°C. Components were separated using a linear gradient of an acetate-formate buffer increasing in concentration in an ACN-ultrapurified H_2_O (80:20) mixture (flow rate 0.3 mL/min). The flow of the liquid chromatography instrument was directed to the mass spectrometer from 2.5 to 5.0 min of the run for detection of acetylcholine [postcolumn makeup flow 50 µL/min (acetonitrile-0.1% FA)]. Mass spectrometric analyses were performed using an API 4000 MS/MS system consisting of an API 4000 dual mass spectrometer detector and a TurboIonSpray interface. Acquisitions were performed in positive ionization mode, with ion spray voltage set at 4.5 kV with a probe temperature of 450°C. The instrument was operated in multiple-reaction-monitoring mode. Data were calibrated and quantified using the Analyst data system (version 1.4.2).

Analysis of adenosine was performed by adding 4 μL of internal standard solution to 20 μL of a 20-fold diluted experimental sample, and then 10 μL were injected into the LC system (Shimadzu Prominence series) by an automated sample injector (SIL-20AD; Shimadzu). Analytes were separated by liquid chromatography using a linear gradient of mobile phase B at a flow rate of 0.200 mL/min on a reversed-phase Atlantis T3 C18 column (2.1 × 150 mm, 3.0-μm particle size; Waters) held at a temperature of 40°C. Mobile phase A consisted of ultra-purified H_2_O with 0.1% FA. Mobile phase B was 75% ACN, 25% ultra-purified H_2_O, and 0.1% FA. Acquisitions were achieved in positive ionization mode using an API 4000 triple quadrupole (Applied Biosystems) equipped with a TurboIonSpray interface. The ion spray voltage was set at 5.5 kV and the probe temperature was 600°C. The collision gas (nitrogen) pressure was held at 6 psi. Data were calibrated and quantified using the Analyst data system (version 1.4.2).

#### Histology.

At least 7 days after the microdialysis experiment, mice were deeply anesthetized with isoflurane, decapitated, and their brains removed. Whole brains were fixed in formalin (10%). Serial coronal sections (35 μm thick) were processed with Perls’ diaminobenzidine (DAB) stain for probe tract detection and counterstained with thionine to reveal cell bodies (Fig. 1*D*). Two investigators blinded to conditions of the microdialysis experiments independently determined the stereotaxic coordinates of each dialysis site (anterior, lateral, and ventral relative to bregma) by comparing the digitized histology sections with a mouse brain atlas (Paxinos and Franklin 2018).

#### Statistical analyses.

The criterion for statistical significance was *P* < 0.05. Stereotaxic coordinates for the dialysis sites are expressed as means ± SD and were used to calculate the three-dimensional Euclidean position of each dialysis site. A two-tailed, unpaired *t* test (GraphPad Prism, v8.3.1; La Jolla, CA) was used to determine whether there was a significant difference between brain sites dialyzed during wakefulness and those dialyzed during isoflurane anesthesia. This procedure was necessary to ensure that differences in neurotransmitter concentrations between wakefulness and anesthesia could not be attributed to differences in brain regions from which the dialysis samples were collected.

Neurotransmitter concentration data were analyzed using Statistical Analysis System (v9.4TS1M5; SAS Institute, Inc.). Neurotransmitter quantification by liquid chromatography-dual mass spectrometry provides measures that are accurate and reliable. Occasionally, measures were lost because an analyte concentration was below the limit of detection, sample impurities disrupted analyte peak detection, or unknown instrument issues prevented analyte detection. The most common reason for missing data in the present study was that the concentration of transmitter in a dialysis sample was below the limit of detection. In such cases, missing data points for individual measures were imputed using lower limit of quantification values obtained from measuring known concentrations of transmitters (i.e., standards). This approach allowed for use of as many data points as possible, thereby increasing both power and precision. Data were considered missing if samples failed to meet quality standards or if inadequate chromatography was performed. Outliers were identified graphically using box and whisker plots, and studentized residual diagnostics. Box and whisker plots of neurotransmitter concentrations were constructed using Microsoft Excel v16.12. Outlier values were neither removed nor imputed.

Nanomolar values for the simultaneously collected neurotransmitters were rank transformed (p. 149, Conover 1999) to compensate for the lack for normally distributed residuals and unequal variances. This nonparametric approach is robust to outliers (p. 117, Conover 1999); thus no outliers were removed from the analyses. A completely randomized design with mixed experimental unit subsampling and corresponding mixed model ANOVA (Little et al. 2006) on ranks was used to test for a main effect of state (wakefulness or isoflurane anesthesia). The Benjamini-Hochberg false discovery rate (fdr) *P*-value adjustment procedure was performed across all tests. Post hoc evaluation of differences in neurotransmitter concentrations during wakefulness vs. isoflurane anesthesia was provided by Fisher’s least significant difference test. Sample sizes were based on previously published, in vivo microdialysis studies of neurotransmitters (Coleman et al. 2006; DeMarco et al. 2004; Douglas et al. 2001, 2002; Flint et al. 2010; Gettys et al. 2013; Van Dort et al. 2009; Wathen et al. 2012) and metabolomics (Bourdon et al. 2018) in B6 mouse. Changes in neurotransmitter concentrations over time were evaluated for each of the eight transmitters by comparing the slopes across the wakefulness sampling period with slopes across the isoflurane sampling period. No a priori statistical power calculations were conducted. Heat maps were produced using Pheatmap (Kolde 2018) (v1.0.12). Pearson correlation network figures were generated using Cytoscape (Shannon et al. 2003) (v3.7.1).

#### Machine learning applied to neurochemical data.

Machine learning is a component of artificial intelligence that trains a computer how to learn. Artificial intelligence is a broader field aiming to develop hardware and software interfaces that emulate human problem solving. Although both approaches date to the 1950s, to the best of our knowledge this study is the first to apply machine learning to evaluate the effects of isoflurane on concentrations of neurotransmitters obtained from the prefrontal cortex. Coexpression networks were created from the time series vectors of each neurotransmitter. A Pearson correlation metric and appropriate thresholds were determined via inspection of the correlation coefficient distribution as well as by random matrix theory. The network topologies of each of the resulting networks from the isoflurane-induced perturbations were compared. The cross-network topological overlap was determined using methods described previously (Weighill and Jacobson 2017).

Random forests (Breiman 2001) is a machine learning method that produces a prediction model and feature importance from bootstrapped decision trees. Iterative random forest (iRF) (Basu et al. 2018) is a method that takes the random forest and adds refinements to determine feature importance. Random intersection trees use the tree structures and determine the importance of feature interactions (Shah and Meinshausen 2014). Here we used iRF leave-one-out prediction (iRF-LOOP) (Cliff et al. 2019), for which the measured neurotransmitters were used as both the predictors and prediction targets in the model. This means that multiple iRF models were built, one to predict the value of each neurotransmitter. The feature set for each model consisted of scalable neurotransmitter concentrations. Another iRF model was built with the prediction target being whether the mouse was awake or anesthetized, with the features being the neurotransmitters. These models were analyzed for feature importance in determining accurate predictions of the given neurotransmitters or of the state of isoflurane anesthesia. The models were trained with 75% of the data, and the other 25% was used to test the accuracy of the model. The iRF figures were produced using Cytoscape (Shannon et al. 2003) (v3.7.1). Application of the forgoing algorithms created models demonstrating prefrontal cortex neurotransmitter networks in control conditions and network reorganization by isoflurane. Whereas Pearson correlations are limited to finding linear relationships, iterative random forest analyses via machine learning can discover both linear and nonlinear relationships. The value of the machine learning approach is further emphasized by the fact that iterative random forest results identify directional, predictive networks in contrast to nondirectional, Pearson-correlative networks.

## RESULTS

### 

#### Isoflurane anesthesia differentially altered neurotransmitter concentrations in prefrontal cortex.

**Fig. 2.**
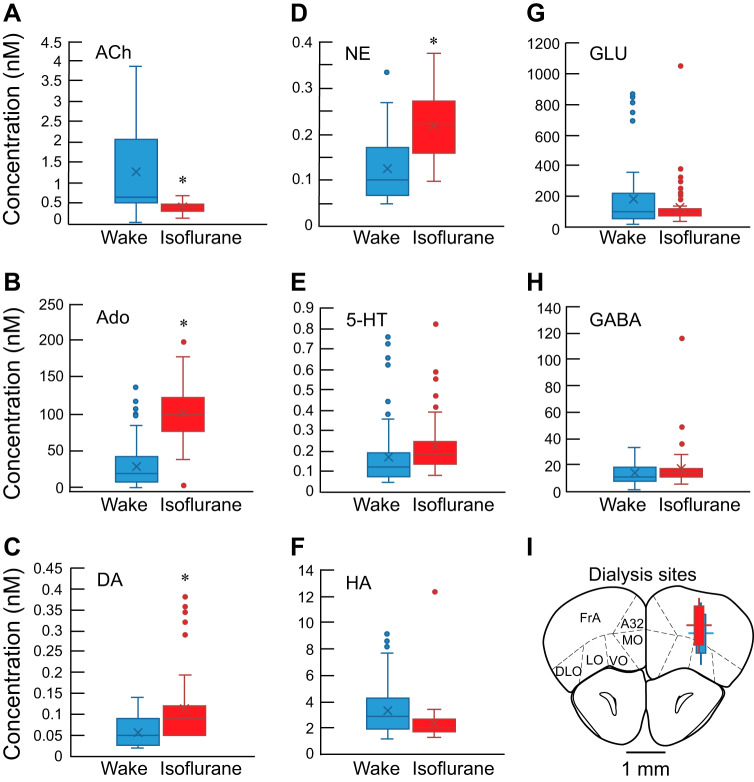
Concentration distributions of 8 neurotransmitters (*A–H*) simultaneously collected during wakefulness (blue; *n* = 12 mice) and isoflurane anesthesia (red; *n* = 11 mice). Concentrations of each transmitter were averaged across the 5 dialysis samples obtained during wakefulness or anesthesia. The *bottom* and *top* of each box indicate the first and third quartiles, respectively. Inside each box, the horizontal line shows the median (second quartile), and the cross (×) inside each box plots the mean. Whiskers indicate the lowest data point within 1.5 times the interquartile range (IQR) of the lower quartile, and the highest data point within 1.5 times IQR of the upper quartile. Outliers are indicated by small dots above and/or below the whiskers. No outliers were removed from the analyses. Asterisks indicate significant (fdr *P* < 0.01) difference from Wake. Neurotransmitters: ACh, acetylcholine; Ado, adenosine; DA, dopamine; GABA, γ-aminobutyric acid; GLU, glutamate; HA, histamine; NE, norepinephrine; 5-HT, serotonin. *I*: summary of the stereotaxic coordinates (means ± SD) of dialysis sites studied during wakefulness (blue cylinder) and isoflurane anesthesia (red cylinder). Cylinders represent dialysis membranes and are drawn to scale (1 mm long, 0.24 mm in diameter) on the bregma 2.77 plate from a mouse brain atlas (Paxinos and Franklin 2018). Cylinders show the mean dialysis probe location. Lines extending from the cylinders indicate SDs in the medial, lateral, dorsal, and ventral dimensions and are drawn to scale. Although the average lateral coordinates for wakefulness and isoflurane experiments were the same, the isoflurane cylinder (red) is offset slightly in the medial direction from the wakefulness cylinder (blue) so that both cylinders are visible. Anatomic areas: A32, cingulate cortex area 32; DLO, dorsolateral orbital cortex; FrA, frontal association cortex; LO, lateral orbital cortex; MO, medial orbital cortex; VO, ventral orbital cortex.

The Fig. 2 box and whisker plots summarize the concentrations of eight neurotransmitters measured during wakefulness (blue) and isoflurane anesthesia (red). Relative to wakefulness, during isoflurane anesthesia the concentration of acetylcholine was significantly lower (Fig. 2*A*; 1.254 ± 1.118 vs. 0.401 ± 0.134 nM; fdr *P* = 0.009) and concentrations of adenosine (Fig. 2*B*; 29.456 ± 29.756 vs. 101.321 ± 38.603 nM; fdr *P* < 0.001), dopamine (Fig. 2*C*; 0.0578 ± 0.0384 vs. 0.113 ± 0.084 nM; fdr *P* = 0.036), and norepinephrine (Fig. 2*D*; 0.126 ± 0.080 vs. 0.219 ± 0.066 nM; fdr *P* = 0.010) were significantly greater. Missing data points (*n* = 97) across the eight measured transmitters comprised 10.5% of the total sample (*n* = 920).

#### Histological confirmation that neurotransmitters were collected from prefrontal cortex.

Figure 2*I* summarizes the average location of microdialysis sites. Average stereotaxic coordinates (mean ± SD relative to bregma; Paxinos and Franklin 2018) for dialysis sites studied during wakefulness (blue cylinder) were 2.6 ± 0.1 mm anterior, 1.4 ± 0.2 mm lateral, and 1.9 ± 0.3 mm ventral. Sites ranged from 2.6 to 2.8 mm anterior, 1.1 to 1.8 mm lateral, and 1.4 to 2.2 mm ventral. During isoflurane anesthesia (red cylinder), dialysis samples were collected from sites that averaged 2.6 ± 0.2 mm anterior, 1.4 ± 0.2 mm lateral, and 1.7 ± 0.2 mm ventral, and ranged from 2.1 to 3.0 mm anterior, 1.0 to 1.7 mm lateral, and 1.4 to 2.0 mm ventral. There was no statistically significant difference in average probe placement between the wakefulness group and the isoflurane group (*t* = 1.446, df = 21, *P* = 0.163). A representative histological section from this study is shown in Fig. 1*D*.

#### Neurotransmitter concentrations across the sampling period.

**Fig. 3.**
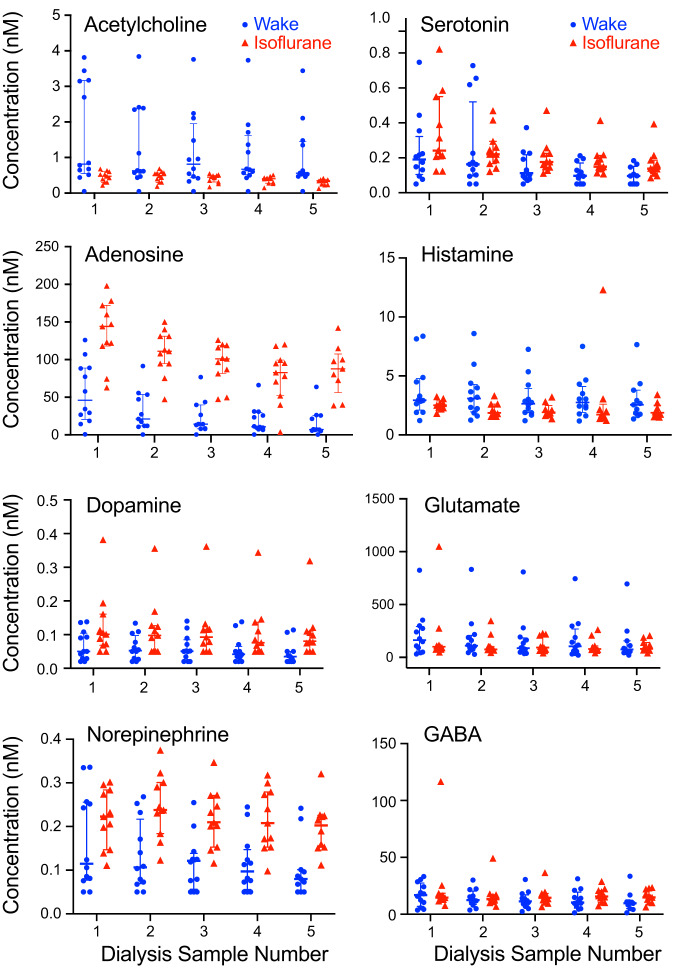
Time course of neurotransmitter concentrations (nM) in dialysis samples collected every 25 min from prefrontal cortex. Using a between-subjects design, 5 sequential dialysis samples were collected from 12 mice during wakefulness (blue circles) and from 11 different mice during isoflurane anesthesia (red triangles). Total sample collection time for each experiment was 125 min. Lines mark median and interquartile range for each dialysis sample.

During in vivo neurochemical sampling, it is critically important to distinguish drug-induced declines in measured analytes from declines in measures that may reflect decreasing dialysis probe recovery, decreasing detector sensitivity, or some unidentified confound. As with previous neurochemical studies in mice (Gettys et al. 2013; Van Dort et al. 2009; Wathen et al. 2012), changes in neurotransmitter concentrations over time were evaluated by comparing the slopes of each neurotransmitter time course during wakefulness and during isoflurane anesthesia. Figure 3 plots the time course for the eight neurotransmitters measured during the five dialysis sampling intervals. Slopes by neurotransmitter during wakefulness and during isoflurane anesthesia, respectively, were as follows: acetylcholine, −0.152 vs. −0.040; adenosine, −8.953 vs. −13.71; dopamine, −0.005 vs. −0.005; norepinephrine, −0.015 vs. −0.008; serotonin, −0.044 vs. −0.042; histamine, −0.201 vs. −0.009; glutamate, −16.23 vs. −18.75; and GABA, −1.388 vs. −1.532. Slope analyses showed that for each neurotransmitter, there was no significant difference between slopes across sampling time comparing wakefulness and isoflurane anesthesia.

#### Isoflurane reconfigured neurotransmitter interactions.

**Fig. 4.**
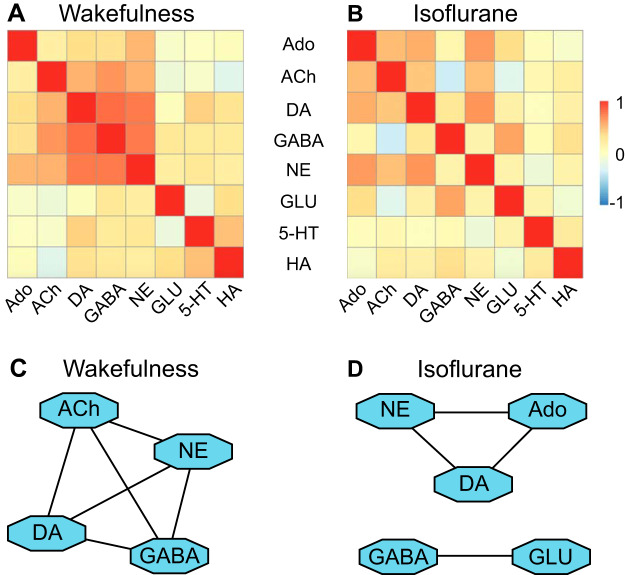
Isoflurane altered neurotransmitter interactions in prefrontal cortex. Heat maps illustrate neurotransmitter interactions during wakefulness (*A*) and isoflurane anesthesia (*B*). The cells in each heat map use color to represent the strength of the pairwise correlations between neurotransmitters (key at *right*). *A*: during wakefulness, high positive correlations between neurotransmitters are visualized by the dark orange cells clustered near the middle of the heat map. *B*: during isoflurane anesthesia, there were fewer high positive correlations than during wakefulness. *C* and *D*: correlation networks were created from Pearson correlation coefficients between neurotransmitters having a correlation value of 0.5 or greater [see Supplemental Table S1 (https://figshare.com/s/6616be980c03708a1c06) and Supplemental Table S2 (https://figshare.com/s/2299b68efc5364a3659e)]. Waking and isoflurane data were analyzed separately. Network nodes (blue octagons) represent neurotransmitters and edges (lines connecting the nodes) indicate high correlations between two transmitters. Connecting lines in the networks reveal differences between wakefulness and isoflurane anesthesia in the transmitters that were coregulated during each state. ACh, acetylcholine; Ado, adenosine; DA, dopamine; GABA, γ-aminobutyric acid; GLU, glutamate; HA, histamine; NE, norepinephrine; 5-HT, serotonin.

The ability to quantify simultaneously collected neurotransmitters made it possible to evaluate relationships among the concentrations of the eight neurotransmitters during wakefulness compared with neurotransmitter concentrations during isoflurane anesthesia. The heat maps in Fig. 4 provide a graphic display of the state-dependent clustering of neurotransmitters by color coding correlations, both positive (red) and negative (blue), between pairs of the eight measured neurotransmitters. During wakefulness (Fig. 4*A*), there were high, positive correlations between acetylcholine and GABA, dopamine and norepinephrine, dopamine and GABA, and GABA and norepinephrine. During isoflurane anesthesia (Fig. 4*B*), there was an overall decrease in high, positive correlations between neurotransmitters. The correlation between acetylcholine and GABA, which was positive during wakefulness, became negative during isoflurane anesthesia. In contrast to wakefulness, there was no positive correlation between dopamine and GABA during isoflurane anesthesia. Concentrations of GABA and glutamate, which were not correlated during wakefulness, became positively correlated during administration of isoflurane (Fig. 4*B*).

The Fig. 4, *C* and *D*, correlation networks show interactions between neurotransmitters with Pearson correlations of 0.5 or greater [Supplemental Table S1; see (https://figshare.com/s/6616be980c03708a1c06) and Supplemental Table S2 (see https://figshare.com/s/2299b68efc5364a3659e)]. During wakefulness (Fig. 4*C*), four neurotransmitters were present in one network, and all of those four transmitters were highly correlated with each other. The multiple lines connecting acetylcholine, dopamine, GABA, and norepinephrine convey those interactions. By contrast, isoflurane anesthesia (Fig. 4*D*) reorganized the neurotransmitters that were highly correlated. Isoflurane anesthesia was characterized by two networks. One isoflurane network comprised norepinephrine, adenosine, and dopamine (Fig. 4*D*). Norepinephrine and dopamine were highly correlated, as they were during wakefulness. However, norepinephrine was no longer highly correlated with acetylcholine or with GABA during isoflurane anesthesia. Instead, adenosine was present in the isoflurane network and was highly correlated with norepinephrine and with dopamine. The second network during isoflurane anesthesia comprised GABA and glutamate (Fig. 4*D*). In contrast to wakefulness, during isoflurane anesthesia GABA was only correlated with glutamate. The data in Fig. 4, *A–D*, reveal a profound reorganization of interactions between neurotransmitters in the prefrontal cortex during isoflurane anesthesia relative to wakefulness.

#### Prediction models.

**Fig. 5.**
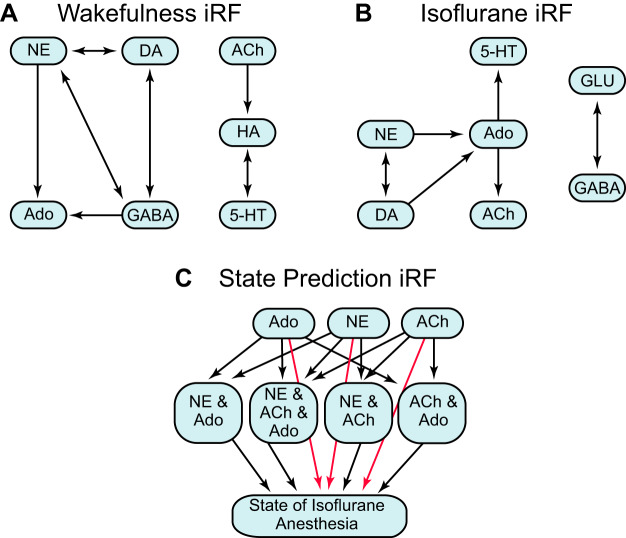
Iterative random forest (iRF) leave-one-out prediction was used to identify neurotransmitters that predict the concentrations of other neurotransmitters in the network. *A* and *B*: blue rounded rectangles (nodes) are connected by arrows (edges) that specify which neurotransmitter concentrations predict other neurotransmitter concentrations. Double-headed arrows identify neurotransmitters with reciprocally predictive concentrations. *C*: this iRF network predicted the behavioral state (wakefulness or isoflurane anesthesia) based on neurotransmitter concentrations. The analysis identified 3 neurotransmitters (*top* row of nodes) that were effective in predicting the state of isoflurane anesthesia, as denoted by the red arrows (edges). The nodes in the *middle* row represent interacting neurotransmitters. Black arrows from the *top* row of nodes to the *middle* row of nodes, and those leading from the *middle* row of nodes, illustrate the interactions among those neurotransmitters that also predicted the state of isoflurane anesthesia. ACh, acetylcholine; Ado, adenosine; DA, dopamine; GABA, γ-aminobutyric acid; GLU, glutamate; HA, histamine; NE, norepinephrine; 5-HT, serotonin.

The iRF-LOOP machine learning algorithm was used to predict higher order interactions among neurotransmitters during wakefulness (Fig. 5*A*) and during isoflurane anesthesia (Fig. 5*B*). When mice were awake (Fig. 5*A*), adenosine, norepinephrine, dopamine, and GABA formed one network in which concentrations of norepinephrine predicted the concentrations of the other three transmitters. Norepinephrine also had reciprocal predictive relationships with dopamine and GABA. A second wakefulness network comprised acetylcholine, histamine, and serotonin. The concentration of acetylcholine predicted the concentration of histamine. Concentrations of serotonin predicted concentrations of histamine, and vice versa. During isoflurane anesthesia (Fig. 5*B*), the wakefulness network defined by adenosine, norepinephrine, and dopamine was altered by the loss of GABA and the addition of acetylcholine and serotonin. GABA and glutamate formed a separate network during isoflurane anesthesia, and the concentrations of these two neurotransmitters were reciprocally predictive.

Figure 5*C* shows the iRF network that was derived from the measured neurotransmitters. iRF was used to predict the state of anesthesia and identified three neurotransmitters with high feature importance: adenosine, norepinephrine, and acetylcholine (*top* row of nodes). The red arrows (edges) originating from the nodes for adenosine, norepinephrine, and acetylcholine indicate that the concentrations of these three transmitters directly predicted the state of isoflurane anesthesia. The iRF and random intersection trees analyses also showed that the same three neurotransmitters in combination (Fig. 5*C*, *middle* row of nodes) predicted the state of isoflurane anesthesia. Overall, this network indicates that the greatest differences between states of wakefulness and isoflurane anesthesia were interactions among adenosine, norepinephrine, and acetylcholine.

## DISCUSSION

Three novel findings emerged from machine learning analyses of these data. First, isoflurane anesthesia caused a reorganization of neurotransmitter interactions in the prefrontal cortex. Second, the state of isoflurane anesthesia was predicted by concentrations of adenosine, norepinephrine, and acetylcholine. Third, specific neurotransmitters were identified that predicted concentrations of other network transmitters. Considered together, the results extend to the neurochemical domain the concept that anesthetics disrupt brain information integration (Alkire et al. 2008; Boly et al. 2017; Golkowski et al. 2019; Li et al. 2019; Suzuki and Larkum 2020; Vlisides et al. 2019). The finding that isoflurane caused reorganization of neurotransmitter networks supports the interpretation that drug-induced changes in neurotransmitter concentrations reflect complex network interactions rather than direct actions of any drug on a single neurochemical cell type.

### 

#### Acetylcholine and adenosine in the prefrontal cortex.

The prefrontal cortex receives cholinergic projections from the basal forebrain (Furster 2015), and the present finding of lower acetylcholine concentrations during isoflurane anesthesia (Fig. 2*A*) agrees with data showing that isoflurane decreases acetylcholine in rat somatosensory cortex (Dong et al. 2006). The lower concentration of acetylcholine during isoflurane anesthesia is consistent with evidence that acetylcholine in rat prefrontal cortex is decreased during the loss of wakefulness caused by sevoflurane, propofol, and physiological sleep (Pal et al. 2016). The present finding of a lower acetylcholine concentration in prefrontal cortex during isoflurane anesthesia than during wakefulness is of particular interest given that dendrosomatic coupling in cortical pyramidal cells is robust during wakefulness and reduced during isoflurane anesthesia (Suzuki and Larkum 2020). This coupling is disrupted by blocking muscarinic cholinergic receptors in cortex of awake mice, suggesting that loss of cholinergic activation of these apical dendrites may play a significant role in anesthesia-induced loss of wakefulness (Suzuki and Larkum 2020).

Adenosine inhibits cell excitability and extracellular levels of adenosine are increased by many classes of general anesthetics (van der Mierden et al. 2018). Adenosine is a sleep-promoting molecule, and extracellular adenosine levels in the cortex rise during prolonged wakefulness (Porkka-Heiskanen and Kalinchuk 2011). Blocking adenosine A1 receptors in prefrontal cortex of B6 mice causes a concentration-dependent increase in prefrontal cortex acetylcholine, concomitant activation of the cortical EEG, and increase in wakefulness (Van Dort et al. 2009). These neurotransmitter changes likely underlie consistent findings that recovery time from isoflurane anesthesia is increased by adenosine agonists (Gettys et al. 2013) and decreased by adenosine antagonist (Fong et al. 2017). The inverse relationship between concentrations of acetylcholine and adenosine in simultaneously collected prefrontal cortex dialysis samples (Fig. 2, *A* and *B*) fits with the previously reported interaction between prefrontal cortex adenosine receptors and acetylcholine levels (Van Dort et al. 2009).

During isoflurane anesthesia, adenosine concentration in the prefrontal cortex was high relative to wakefulness (Fig. 2*B*). Adenosine is a product of cellular energy metabolism, and cortical adenosine increases during sleep deprivation and decreases during non-rapid eye movement (NREM) sleep (Porkka-Heiskanen and Kalinchuk 2011). These relationships suggest that adenosine is a biomarker of homeostatic sleep need (Greene et al. 2017). The present finding of a higher adenosine concentration in prefrontal cortex during isoflurane anesthesia relative to wakefulness is consistent with evidence that isoflurane anesthesia does not satisfy the homeostatic need for NREM sleep (Zhang et al. 2016).

#### Dopamine and norepinephrine concentrations in prefrontal cortex were greater during isoflurane anesthesia than during wakefulness.

Reciprocal connections between the prefrontal cortex and ventral tegmental area, raphe nuclei, and locus coeruleus provide the prefrontal cortex with dopamine, serotonin, and norepinephrine, respectively (Dembrow and Johnston 2014). Dopamine has an arousal-promoting role relevant to anesthesia (Solt et al. 2014) and sleep (Brown 2016). The results show that dopamine in the prefrontal cortex of B6 mouse was greater during isoflurane anesthesia than during wakefulness (Fig. 2*C*). Dopamine is a precursor of norepinephrine, and the finding that the concentration of norepinephrine in the prefrontal cortex was greater during isoflurane anesthesia than during wakefulness (Fig. 2*D*) is consistent with previous studies in rat. Norepinephrine in the prefrontal cortex was greater during administration of anesthetic doses of isoflurane (Kushikata et al. 2005), ketamine, propofol, and midazolam (Kubota et al. 1999), and by xenon and nitrous oxide (Yoshida et al. 2010). The functional relevance of greater norepinephrine concentrations in the prefrontal cortex during administration of anesthetic and sedative/hypnotic drugs is unclear relative to the well-recognized association between wakefulness and increased activity of monoaminergic neurons (Brown et al. 2012). One interpretation is to acknowledge that monoaminergic systems act subcortically to promote arousal (Brown et al. 2012; Solt et al. 2014) and that additional transmitters such as orexin (Mieda 2017) and histamine (Brown et al. 2012) also promote wakefulness. Concurrent inhibition of all arousal systems in all brain regions is not necessary for anesthetic-induced loss of wakefulness (Gompf et al. 2009), and different wakefulness promoting systems have been shown to produce differing traits of arousal during the state of isoflurane anesthesia in rat (Kenny et al. 2016).

#### Unanticipated findings during isoflurane anesthesia.

Relative to wakefulness, concentrations of serotonin, histamine, glutamate, and GABA were not significantly altered during isoflurane anesthesia (Fig. 2, *E–H*). Serotonin, histamine, and glutamate were not present in the iRF networks during wakefulness (Fig. 5*A*) and were not predictors of the state of anesthesia (Fig. 5*C*). These unanticipated findings suggest that prefrontal cortex concentrations of these three transmitters may be less important for determining states of wakefulness or loss of wakefulness than adenosine, norepinephrine, and acetylcholine.

#### Reconfiguration of neurotransmitter interactions during isoflurane anesthesia.

During general anesthesia, the entire nervous system is exposed to anesthetic agents. Thus quantifying multiple neurotransmitters is more likely to represent state-dependent changes in the neurochemical milieu than is measuring one neurotransmitter at a time. Correlation analyses between pairs of transmitters revealed an isoflurane-induced decrease in positive correlations (Fig. 4, *A* and *B*) and a reorganization of interactions between cortical transmitters (Fig. 4, *C* and *D*). The iRF analyses demonstrated that highly correlated transmitters predicted the concentrations of other network transmitters (Fig. 5, *A* and *B*). Machine learning further identified three transmitters that predicted that state of isoflurane anesthesia directly, as well as by interacting with each other (Fig. 5*C*). None of these relationships could have been revealed by measuring transmitters that were collected one at a time. These novel findings are consistent with the view that interactions among adenosine, norepinephrine, and acetylcholine play a key role in modulating levels of cortical and behavioral arousal.

#### Limitations.

The rodent prefrontal cortex comprises multiple subregions (Gretenkord et al. 2017) which have been defined based on cytoarchitecture, connectivity, and/or functionality (Carlén 2017). The present results were obtained mainly from the frontal association cortex (Fig. 2*I*, FrA), as defined in a mouse brain atlas (Paxinos and Franklin 2018). The results do not imply a singular role for the prefrontal cortex in regulating states of consciousness. Rather, the present results demonstrate the potential for unique insights to emerge from future studies designed to measure neurotransmitters and metabolites simultaneously from two or more brain regions. Furthermore, this study was limited to quantifying the effects of only one concentration of isoflurane. The findings encourage future studies specifically designed to administer different doses and classes of general anesthetics. Testing anesthetic doses greater than the EC_50_ may decrease variability in neurotransmitter concentration. Such studies have exciting potential to enhance neurochemical phenotyping unique to sex, species, and genotype. An additional complexity is that although measured changes in neurotransmitter concentrations were likely caused by isoflurane-induced neuronal hyperpolarization (Ries and Puil 1999), changes in breathing are known to produce significant changes in human prefrontal cortical function (Heck et al. 2019; Thomas et al. 2005). None of the anesthetized mice in the present study exhibited significant bradypnea, but subanesthetic concentrations of isoflurane can decrease the hypercapnic ventilatory response in B6 mice (Massey and Richerson 2017). Core body temperature was maintained in the anesthetized mice, yet isoflurane can cause a decrease in brain temperature (Shirey et al. 2015). Thus the isoflurane-induced changes in neurotransmitter concentrations may have been caused by neuronal hyperpolarization within the prefrontal cortex and modulated by changes in breathing and/or brain temperature. Arguing against such modulation is the finding that the slopes of the neurotransmitter concentrations across time (Fig. 3) were not significantly different between wakefulness and isoflurane anesthesia.

#### Conclusions.

The results show that assessing neurotransmitter interactions in prefrontal cortex using machine learning can differentiate and predict the state of isoflurane anesthesia. These findings also imply that any conclusion about a systemically delivered anesthetic exerting direct effects on the concentration of any single transmitter is oversimplified. Likewise, a single measure of cortical EEG connectivity is unlikely to be a reliable correlate of surgical anesthesia (Vlisides et al. 2019). The present results support the conclusion that isoflurane caused a dynamic reconfiguration of complex and interacting neurotransmitter networks. Achieving a functional connectomics perspective of the brain (Fornito et al. 2015) must ultimately incorporate neurotransmitter data.

Machine learning approaches outperform humans in speed and efficiency with which patterns can be detected in very large data sets. The present results encourage application of these analytic techniques to multiple brain regions, to neuroendocrine molecules (Jiang-Xie et al. 2019), and to molecules comprising the brain metabolome (Bourdon et al. 2018). Over time, the systematic curation of such measures will provide building blocks needed to construct a chemical cartography underlying wakefulness and anesthesia.

## GRANTS

This work was supported by National Natural Science Foundation of China Grant NSFC-8167103 (W.M.) and National Key Research and Development Program of China Grant 2018YFC2001900 (W.M.), The Chinese Scholarship Council, Beijing, China (X.Z.), and the Departments of Anesthesiology and Psychology, University of Tennessee, Knoxville, TN. Funding for the code development for iterative random forests was provided by The Center for Bioenergy Innovation. US Department of Energy (DOE) Bioenergy Research Centers are supported by the Office of Biological and Environmental Research in the DOE Office of Science.

## DISCLAIMERS

This manuscript has been co-authored by UT-Battelle, LLC under Contract No. DE-AC05-00OR22725 with the US Department of Energy. The United States Government retains and the publisher, by accepting the article for publication, acknowledges that the United States Government retains a non-exclusive, paid-up, irrevocable, worldwide license to publish or reproduce the published form of this manuscript, or allow others to do so, for United States Government purposes. The Department of Energy will provide public access to these results of federally sponsored research in accordance with the DOE Public Access Plan (https://energy.gov/downloads/doe-public-access-plan).

## DISCLOSURES

No conflicts of interest, financial or otherwise, are declared by the authors.

## AUTHOR CONTRIBUTIONS

R.L. and H.A.B. conceived and designed research; X.Z. and A.G.B. performed experiments; X.Z., A.G.B., J.M.P., P.C.J., B.J.G., J.R., A.M.C., J.B.B., D.A.J., R.L., and H.A.B. analyzed data; X.Z., A.G.B., J.M.P., P.C.J., B.J.G., J.R., W.M., J.B.B., D.A.J., R.L., and H.A.B. interpreted results of experiments; X.Z., A.G.B., B.J.G., J.R., D.A.J., R.L., and H.A.B. prepared figures; D.A.J., R.L., and H.A.B. drafted manuscript; X.Z., A.G.B., J.M.P., P.C.J., B.J.G., J.R., A.M.C., W.M., J.B.B., D.A.J., R.L., and H.A.B. edited and revised manuscript; X.Z., A.G.B., J.M.P., P.C.J., B.J.G., J.R., A.M.C., W.M., J.B.B., D.A.J., R.L., and H.A.B. approved final version of manuscript.
